# Decreasing tendon rupture and tenolysis with wide awake surgery

**DOI:** 10.1186/1753-6561-9-S3-A66

**Published:** 2015-05-19

**Authors:** Donald Lalonde

**Affiliations:** 1 Department of Plastic and Reconstructive Surgery, Dalhousie University, Saint John, New Brunswick, Canada, E2K 1J5

## 

There are 5 main reasons that I never want to repair a flexor tendon in sedated patients ever again. If a patient is wide awake (unsedated) with no tourniquet and only lidocaine with epinephrine is used in the fingers and hand for local anesthesia and hemostasis, 5 major things happen to improve the results:

**1) Decrease rupture rate with intraoperative active testing of repair**: Injection of only lidocaine with epinephrine wherever incisions will be made in the finger and hand permits comfortable tourniquet-free awake patients cooperate with active full flexion and extension of fingers testing during the surgery. The rupture rate decreases because the repair can be tested with full active flexion and full active extension to make sure that there is no gapping which will lead to rupture. If there is gapping, the repair can be augmented and the gap eliminated by the surgeon before the skin is closed so there will be no rupture. This has decreased the rupture rate by 7% in the largest WALANT flexor tendon repair series to date (Higgins A, Lalonde DH, et al. Avoiding flexor tendon repair rupture with intraoperative total active movement examination. Plast Reconstr Surg 2010;126(3):941.)

**2) Knowing there is no gap with a full fist permits half a fist true active movement post op for better results**: If the surgeon sees the patient make a **full** fist and completely extend the fingers with no gapping during the surgery, he will be confident to let the patient start controlled TRUE active movement 3 days after the surgery (not Kleinert rubber bands nor “place and hold”). True active movement means **up to half** a fist of active flexion and extension. If the patient can make a full fist at surgery, he is not likely to rupture with half a fist after surgery.

**3) Decrease tenolysis rate by testing active movement intraoperatively:** The awake cooperative pain free patient can make a complete fist to make sure the repair fits through the pulleys before the surgeon closes the skin. If the repair does not fit at the time of surgery, it will not fit later and the surgeon will need to come back and do a tenolysis. The distal half of A2, and the entire A4 pulleys can be vented (divided) without major bowstringing if adjacent cruciate pulleys are maintained. Only the pulleys that need be vented are vented. Active movement during the surgery reveals what needs to be done to decrease the tenolysis rate.

**4) Knowing what happens to active movement if superficialis is repaired during surgery:** If the active movement seen during surgery is downgraded by repairing superficialis, the superficialis repair can be taken down, and a slip of superficialis can be resected if required to optimize movement.

**5) An educated flexor tendon repair patient who has seen his finger during surgery is more likely to be cooperative after surgery:** The surgeon (and therapist in our institution) gets a minimum of 90 minutes to educate the patient during surgery about how to get a good result after surgery. Seeing is believing. A patient who has seen the challenges involved in repairing the tendon at surgery has much more of a vested interest and a better understanding of how to cooperate to get a good result.

